# Neuropsychological Impact of West Nile Virus Infection: An Extensive Neuropsychiatric Assessment of 49 Cases in Canada

**DOI:** 10.1371/journal.pone.0158364

**Published:** 2016-06-28

**Authors:** Zainab Samaan, Stephanie McDermid Vaz, Monica Bawor, Tammy Hlywka Potter, Sasha Eskandarian, Mark Loeb

**Affiliations:** 1 Department of Psychiatry & Behavioral Neurosciences, McMaster University, Hamilton, Ontario, Canada; 2 Department of Clinical Epidemiology & Biostatistics, McMaster University, Hamilton, Ontario, Canada; 3 Population Genomics Program, Chanchlani Research Centre, McMaster University, Hamilton, Ontario, Canada; 4 Cleghorn Early Intervention in Psychosis Program, St. Joseph’s Healthcare, Hamilton, Ontario, Canada; 5 MiNDS Neuroscience Graduate Program, McMaster University, Hamilton, Ontario, Canada; 6 Division of Infectious Diseases, McMaster University, Hamilton, Ontario, Canada; 7 Department of Pathology & Molecular Medicine, McMaster University, Hamilton, Ontario, Canada; Washington University, UNITED STATES

## Abstract

**Background:**

West Nile virus emerged as an important human pathogen in North America and continues to pose a risk to public health. It can cause a highly variable range of clinical manifestations ranging from asymptomatic to severe illness. Neuroinvasive disease due to West Nile virus can lead to long-term neurological deficits and psychological impairment. However, these deficits have not been well described. The objective of this study was to characterize the neuropsychological manifestations of West Nile virus infection with a focus on neuroinvasive status and time since infection.

**Methods:**

Patients from Ontario Canada with a diagnosis of neuroinvasive disease (meningitis, encephalitis, or acute flaccid paralysis) and non-neuroinvasive disease who had participated in a cohort study were enrolled. Clinical and laboratory were collected, as well as demographics and medical history. Cognitive functioning was assessed using a comprehensive battery of neuropsychological tests.

**Results:**

Data from 49 individuals (32 with West Nile fever and 17 with West Nile neuroinvasive disease) were included in the present cross-sectional analysis. Patterns of neuropsychological impairment were comparable across participants with both neuroinvasive and non-neuroinvasive West Nile virus infection on all cognitive measures. Neuropsychiatric impairment was also observed more frequently at two to four years post-infection compared to earlier stages of illness.

**Conclusions:**

Our data provide objective evidence for cognitive difficulties among patients who were infected with West Nile virus; these deficits appear to manifest regardless of severity of West Nile virus infection (West Nile fever vs. West Nile neuroinvasive disease), and are more prevalent with increasing illness duration (2–4 years vs. 1 month). Data from this study will help inform patients and healthcare providers about the expected course of recovery, as well as the need to implement effective treatment strategies that include neuropsychological interventions.

## Introduction

West Nile Virus (WNV) has emerged as an important human pathogen in North America. The virus was first isolated from the blood of a febrile patient in northern Uganda in 1937 [1]. Outbreaks of West Nile fever and meningoencephalitis have since been described in many parts of the world, including Africa, Europe, the Middle East, and Asia [2]. The first North American outbreak was in 1999 in New York City [3], where 61 cases of WNV meningoencephalitis were reported. Since then there has been a dramatic increase in the incidence of human cases of WNV in North America [4]. In 2007, there were 2,353 cases reported in Canada, the highest number reported annually since WNV was first detected in 2002 [5].

The pathological changes within the central nervous system due to WNV, an enveloped RNA virus, appear to be due to several factors, including the direct result of viral proliferation within neuronal and glial cells, cytotoxic immune response to infected cells, diffuse perivascular inflammation, and microglial nodule formation [6, 7]. The range of clinical manifestations of WNV infection is however highly variable. Of infected persons, approximately 20% develop mild symptoms (“West Nile Fever” e.g., fever, malaise, headache, myalgia, rash), and about 1% develop West Nile neuroinvasive disease (WNND) (e.g. meningitis, encephalitis, or acute flaccid paralysis) [8–11]. The incidence of severe neurological syndromes increases with age [9]. Although less common, other syndromes include peripheral neuropathy, polyradiculopathy, and optic neuritis [12, 13].

Recent studies of persons infected with West Nile virus report that symptoms and signs, such as fatigue, psychological dysfunction, and motor abnormalities, can persist for months after symptom onset [14–24]. Existing reports provide valuable information on self-reported outcomes [14–23], but have limitations including lack of validated instruments to measure neuropsychological functioning [14–19, 22–24].

Despite a finding of a favourable prognosis with respect to health returning to pre-morbid level [25], many patients report persistent somatic and psychological complaints, including difficulties concentrating, problems with word-finding, and sense of poor general health [19, 26]. These findings are based on cross-sectional studies using a broad battery of neuropsychological tests. Although these reports provide important insight, they are limited because they do not provide a detailed characterization of patients’ cognitive and psychiatric symptoms. While current reports show that those with WNND are more likely to report neurological deficits [27] and more severe depression symptoms [28], these studies are limited by both small sample sizes and their cognitive characterization [28]. Moreover, significant depressive symptoms several years after the WNV infection have been reported [29] but there are limited data on cognitive function following WNV infection. A small study of 22 patients with WNV infection from Greece showed cognitive impairment, subjective weakness and depressive symptoms, however the study measures were not reported and formal neuropsychiatric testing was not performed [30].

In the current study, we provide a comprehensive assessment of neuropsychological manifestations of WNV infection including characterization of WNV infection with clinical, laboratory and neuroimaging confirmation. We specifically aim to (1) characterize the neuropsychological (NP) manifestations of WNV infection with emphasis on differences between WNND (meningitis, encephalitis and meningo-encephalitis) and WNV fever; (2) investigate the NP manifestations at different times since infection using cross-sectional design. We hypothesize that WNV infection is associated with impaired cognitive function irrespective of severity of WNV illness (WNND or WNV fever), which is based on anecdotal experience where there does not appear to be clear cut patterns of NP manifestations based on severity.

## Methods

### Eligibility criteria

Patients with a diagnosis of neuroinvasive disease (meningitis, encephalitis, or acute flaccid paralysis) and non-neuroinvasive disease who had participated in a previous cohort study [25] were eligible. For neuroinvasive disease we employed diagnostic criteria used by the Centers for Disease Control and Prevention, as described by Sejvar et al. (Box 1) [14]. Since complications of West Nile virus infection in children are generally less frequent, we excluded persons <18 years [31].

**Box 1. Definition of West Nile virus classifications, as presented in Sejvar et al. 2003**

*Caption*: Adapted from Sejvar et al. Neurologic manifestations and outcome of West Nile virus infection. *JAMA*. *Volume 290*, edn. United States; 2003: 511–515.

**Definition of Cases** (as classified by Sejvar et al.)

**West Nile Meningitis**
1. Clinical signs of meningeal inflammation, including nuchal rigidity, Kernig or Brudzinski sign, or photophobia or phonophobia
2. Additional evidence of acute infection, including 1 or more of the following: fever (>38°C) or hypothermia (<35°C); cerebrospinal fluid pleocytosis (≥ 5 leukocytes/mm3); peripheral leukocyte count >10 000/mm3; neuroimaging findings consistent with acute meningeal inflammation

**Encephalitis**
1. Encephalopathy (depressed or altered level of consciousness, lethargy, or personality change lasting ≥24 hours)
2. Additional evidence of central nervous system inflammation, including 2 or more of the following: fever (≥38°C) or hypothermia (≤35°C); cerebrospinal fluid pleocytosis (≥5 leukocytes/mm3); peripheral leukocyte count >10 000/mm3; neuroimaging findings consistent with acute inflammation (with or without involvement of the meninges) or acute demyelination; presence of focal neurologic deficit; meningismus (as defined in A); electroencephalography findings consistent with encephalitis; seizures, either new onset or exacerbation of previously controlled

**Acute Flaccid Paralysis**
1. Acute onset of limb weakness with marked progression over 48 hours
2. At least 2 of the following: asymmetry to weakness; areflexia/hyporeflexia of affected limb(s); absence of pain, paresthesia, or numbness in affected limb(s); cerebrospinal fluid pleocytosis (≥5 leukocytes/mm3) and elevated protein levels (≥45 mg/dL); electrodiagnostic studies consistent with an anterior horn cell process; spinal cord magnetic resonance imaging documenting abnormal increased signal in the anterior gray matter

We also excluded those with pre-morbid conditions that would likely impact neuropsychological testing including: diagnosis of dementia or mild cognitive impairment, documented persistent cognitive deficits due to head trauma, or intellectual disabilities. This study has been approved by the Hamilton Integrated Research Ethics Board (HIREB) and written informed consent was obtained from participants.

### Depression and Fatigue

As previously reported, we used the Depression Anxiety Stress Scale (DASS) [32, 33], and the Fatigue Severity Score [34] to capture depressive symptoms and persistent fatigue [25]. The DASS depression scale is a 14-item scale that includes an assessment of dysphoria, lack of interest or involvement. Scores were summed with a possible range from 0 (no symptoms) to 42 and further classified by severity as follows: 1. Depression: Normal (0–9), Mild (10–13), Moderate (14–20), Severe (21–27), Extremely Severe (28+); 2. Anxiety: Normal (0–7), Mild (8–9), Moderate (10–14), Severe (15–19), Extremely Severe (20+); 3. Stress: Normal (0–14), Mild (15–18), Moderate (19–25), Severe (26–33), Extremely Severe (34+). The Fatigue Severity Scale measures the perceived level of fatigue using a Likert scale where the score ranges from 1 (low fatigue level) to 7 (high fatigue level). Normative data by Grace et al. 2006 indicate that the mean in a healthy population (n = 16) is 2.3 (SD 0.7) [35].

### Neuropsychological Outcomes

A comprehensive battery of neuropsychological tests was administered to each participant. The following domains of cognitive functioning were assessed: verbal learning and memory, visual learning and memory, executive functioning, attention and concentration, speed of information processing, visuospatial functioning, and fine motor skills. A symptom validity measure was included as a means of gauging adequate “cognitive effort” or “engagement” during the assessment [36]. Tests were selected to represent the domains of cognitive functioning to be studied and were consistent with the consensus of published test compendia [37, 38].

#### Symptom Validity

The Test of Memory Malingering (TOMM) is a symptom validity test which is frequently used to assess whether a participant is putting forth adequate “cognitive effort” during an assessment [39]. There are two initial trials in which 50 pictorial stimuli are presented, and then the participant is asked to identify the correct item from a two-alternative memory display. An optional Retention Trial is given if the participant fails to meet a criterion of 45 correct responses on Trial 2. Insufficient cognitive effort is suspected if the participant fails to meet the criterion of 45 correct items either on Trial 2 or the Retention Trial [39]. The TOMM has been validated in varied neurological populations [40, 41] and is the most frequently used measure of symptom validity among neuropsychologists with special expertise in the area [42].

#### Verbal Learning and Memory

The Hopkins Verbal Learning Test-Revised (HVLT-R) is a list learning and memory test that is brief, easy to administer, and tolerable for a variety of clinical populations [43]. A 12-item list is presented orally for three learning trials and immediately following each presentation, the participant is asked to recall as many words from the list as possible. A delayed free recall trial (trial 4) occurs 20 to 25 minutes later, followed by a recognition trial. The HVLT-R has six alternate forms that make it ideal for repeat testing. Research has demonstrated the reliability and construct validity of the standard learning and recall measures [44–46].

#### Visual Learning and Memory

The Brief Visuospatial Memory Test-Revised (BVMT- R) is a measure of visual-spatial learning and memory for a matrix of six abstract figures [47]. The figures are held before the participant for 10 seconds and then the participant is asked to reproduce the designs using paper and pencil. There are three free-recall trials, a delayed recall trial after 20 to 25 minutes, and a recognition memory trial. There are six equivalent alternate forms for repeat administrations with minimal practice effects [48]. Studies have shown that the BVMT-R is a useful measure for detecting memory impairment in a variety of neurological and neuropsychiatric conditions [49, 50].

#### Executive Functioning

The Tower of London-DX 2nd Edition (TOL-DX) is a standardized measure of problem-solving, planning, and visuospatial working memory abilities [51]. The examiner uses one tower and a set of beads to display the desired goal and the participant rearranges a second set of beads on a second tower to match the examiner’s configuration. There is a planning element because the success of the task relies on a predetermined set of correctly executed steps and responses. The TOL has been used to examine a number of clinical disorders including Huntington’s disease [52], Parkinson’s disease [53], and autism [54]. The Controlled Oral Word Association Test (COWA) is a widely used measure of verbal fluency [55]. In successive one-minute trials, the participant is asked to generate as many words as possible beginning with each of three designated letters (excluding proper nouns or variations of a previously said word). The COWA has good reliability and has demonstrated validity for identifying executive cognitive impairment and recovery [56, 57].

#### Attention

The Stroop Color and Word Test (SCWT) is a well established measure of selective attention and response inhibition [58–60]. The participant is required to inhibit competing information while making automatic reading responses in order to maintain attention on the target stimuli. It is a brief test consisting of three trials, each with a 45 second time limit. The first trial requires the participant to quickly read a page of color words printed in black ink. For the second trial, the participant names the color hues printed as a series of “Xs”, and the third trial requires the participant to name the color hues printed as competing color words (e.g., “red” printed in blue ink). An association between performance on the SCWT and frontal lobe activation has been demonstrated in a number of neuroimaging and electrophysiological studies [61, 62].

WAIS-III: Letter-Number-Sequencing (LNS) is a commonly used measure of working memory (i.e., information storage with manipulation) [63]. It is brief, easy to administer, and has been used with participants presenting with a wide range of clinical conditions. The participant is read a mixed series of numbers and letters and then asked to repeat the sequence, naming the numbers first in ascending order followed by the letters in alphabetical order. Factor analyses have consistently shown that this test requires working memory skills [63, 64].

WAIS-III: Digit Span (DS) is a brief measure of attention, concentration, and working memory [63]. The participant is required to recall sequences of numbers that increase in length, first in the order they are presented and then in the reverse order of presentation. Research has shown that it is sensitive to establishing and sustaining a focus of attention [65].

#### Speed of Information Processing

The Symbol Digits Modalities Test (SDMT) is a speed of information processing task requiring complex scanning and visual tracking [37, 66]. Although both written and oral versions are available, the oral-response administration will be used in this study to eliminate the possibility that motor difficulties secondary to WNV infection might contribute to poorer performance. A series of nine symbols are paired with a single digit in a key at the top of a sheet of paper, with the remainder of the page presenting a randomized sequence of symbols. The participant is required to respond by voicing the digit associated with each symbol as quickly as possible within a 90 second time limit. The SDMT is extremely sensitive to brain insults in adults and has been used with a variety of clinical populations [38, 67, 68].

#### Visual Perception

The Judgment of Line Orientation test (JLO) is a frequently used measure of spatial perception and orientation [69]. The participant is required to visually match two line segments of varying spatial orientation to a set of longer lines arranged in a semicircle on a response card. The JLO is a reliable and valid measure [55, 56] and research has demonstrated that the measure taps spatial perceptual abilities independent of other cognitive abilities [70].

#### Motor Function

The Grooved Pegboard (GP) is a well-established test of motor speed and fine manual dexterity [71]. The participant is required to insert 25 cylindrical pegs successively and as quickly as possible into keyhole-shaped holes which are ordered in a 5 x 5 matrix. The first trial is completed using the dominant hand, and the second trial is completed with the non-dominant hand.

The study research nurse was trained by a clinical neuropsychologist to administer the battery of tests described above to each participant according to the standardized administration instructions for each instrument.

### Statistical Methods

We present descriptive statistics of the participants in this study; mean (standard deviation, SD) were computed for continuous variables, including demographics and illness characteristics, and frequency of any impairment among the sample was presented as number (percent) of participants. We provide univariate correlations to detect any differences between the two groups using Pearson’s chi-square for categorical variables and student’s t-test for continuous variables, with the alpha set to 0.0019 to adjust for multiple comparisons. We performed all statistical analysis using STATA Version 12 (StataCorp LP, College Station, USA). The study is reported in adherence to the Strengthening the Reporting of Observational Studies in Epidemiology (STROBE) statement [72].

## Results

**Fig 1:**
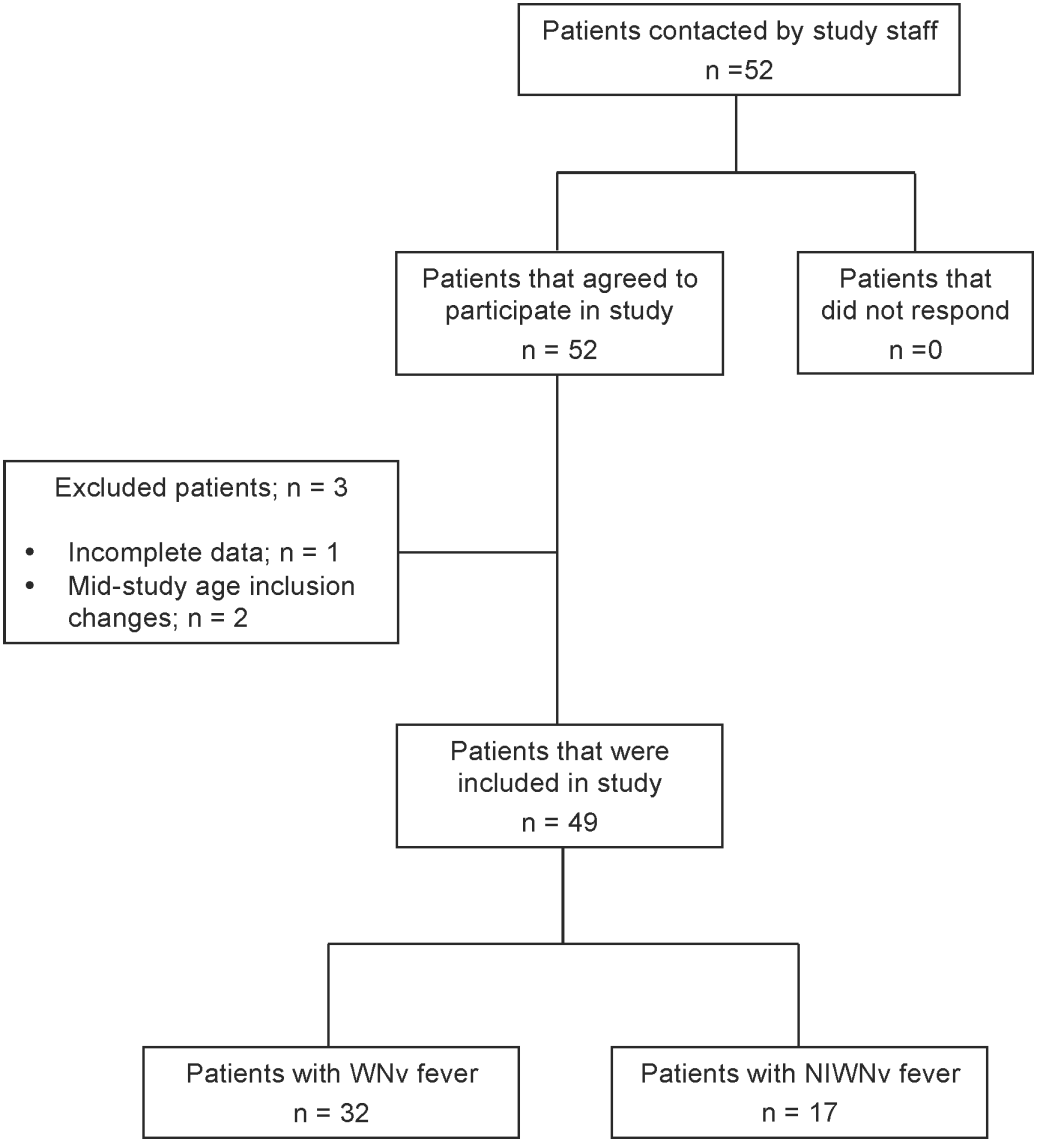
Flow of participants included in study. Participant inclusion at each stage of recruitment is outlined and reasons for exclusion are described.

Data from 49 individuals (mean age = 51.47 years, SD = 11.48) were included in the present analysis (Fig 1). Our sample included 31 females and 18 males. WNND was observed among 17 participants and classified as West Nile encephalitis (n = 7), West Nile meningitis (n = 1), West Nile meningo-encephalitis (n = 9). The remainder of participants (n = 32) were classified as having non-neuroinvasive WNV fever. Mean duration of illness in months was 22.3 (SD 16.1) and 20.5 (SD 11.3) for WNV and WNND, respectively. DASS and FSS scores for depression, anxiety, stress, and fatigue were within normal ranges and were similar between groups. The demographic, illness, and clinical characteristics of the participants are presented in Table 1 by neuroinvasive status; there were no differences between patient groups among these characteristics.

**Table 1:** Sample demographics and illness characteristics.

Variable	WNV n = 32	WNND n = 17	p<0.0019^a^
Age (years); mean (SD)	52.0 (8.9)	50.5 (15.5)	0.662
Men; n (%)	12 (37.5)	6 (35.3)	0.882
Years of education; mean (SD)	13.2 (2.6)	13.7 (3.9)	0.605
Duration of illness (months); mean (SD)	22.3 (16.1)	20.5 (11.3)	0.651
DASS Depression score; mean (SD)	5.0 (7.4)	6.9 (9.1)	0.476
DASS Anxiety score; mean (SD)	3.4 (4.4)	6.1 (6.6)	0.095
DASS Stress score; mean (SD)	7.1 (6.9)	11.2 (10.4)	0.111
Fatigue severity score; mean (SD)	2.9 (2.0)	3.3 (1.8)	0.555

WNV: West Nile virus; WNND: West Nile Virus neuroinvasive disease; SD: standard deviation; DASS: Depression Anxiety Stress Scale

^a^Univariate analysis between WNV and WNND groups; Bonferroni-adjusted alpha for statistical significance is 0.0019

**DASS Severity Classifications:** Depression: Normal (0–9), Mild (10–13), Moderate (14–20), Severe (21–27), Extremely Severe (28+); Anxiety: Normal (0–7), Mild (8–9), Moderate (10–14), Severe (15–19), Extremely Severe (20+); Stress: Normal (0–14), Mild (15–18), Moderate (19–25), Severe (26–33), Extremely Severe (34+)

**FSS:** Normative data by Grace et al. 2006 indicate that the mean in a healthy population (n = 16) is 2.3 (SD 0.7)

Results from the neuropsychological test classifications are presented in Table 2. The findings for the entire sample of participants (both WNV fever and WNND) indicate that a substantial number of patients demonstrated impairment on any measures of: motor functioning (28%-42%), verbal learning and memory (11%-36%), and some measures of executive functioning (11%-30%). A smaller proportion of patients were impaired on tests of visual learning and memory (3%-24%), information processing speed (6%-18%), visual-spatial ability (3%-6%), and attention (0%-12%). The pattern of neuropsychological test findings was generally consistent between groups, with the exception of a few measures. Although a greater proportion of patients with WNND showed impairment in verbal learning and memory (BVMT-R Delayed Recall) and executive functioning (TOL—Rule Violation, TOL—Execution time) compared to patients with WNV, these results did not remain significant after correcting for multiple testing error (Table 2).

**Table 2:** Number of patients showing any impairment on neuropsychological assessments.

Assessment	WNV n = 32	WNND n = 17	p<0.0019^a^
	n (%)	n (%)	
Verbal Learning and Memory			
HVLT-R Total Recall	9 (28.1)	6 (35.3)	0.818
HVLT-R Delayed Recall	9 (28.1)	4 (23.5)	0.604
HVLT-R Delayed Recognition	8 (25.0)	2 (11.8)	0.315
Visual Learning and Memory			
BVMT-R Total Recall	2 (6.3)	3 (17.6)	0.210
BVMT-R Delayed Recall	1 (3.1)	4 (23.5)	0.025
BVMT-R Delayed Recognition	5 (15.6)	4 (23.5)	0.496
Executive Functioning			
COWAT	10 (31.3)	5 (29.4)	0.894
Semantic Fluency	4 (12.5)	3 (17.6)	0.624
TOL—Rule Violation	0 (0)	2 (11.8)	0.037
TOL—Time violation	7 (21.9)	5 (29.4)	0.601
TOL—Execution time	1 (3.1)	4 (23.5)	0.025
TOL—Problem solving time	4 (12.5)	5 (29.4)	0.146
Information Processing Speed			
SDMT	2 (6.3)	3 (17.6)	0.210
Attention			
Stroop Interference	2 (6.3)	2 (11.8)	0.502
LNS	0 (0)	1 (5.9)	0.166
Digit Span	0 (0)	0 (0)	n/a
Visual-spatial Ability			
JLO	1 (3.1)	1 (5.9)	0.642
Fine Motor Skills			
Grooved—Dominant hand	10 (31.3)	7 (41.2)	0.487
Grooved—Non-dominant hand	9 (28.1)	6 (35.3)	0.604

WNV: West Nile virus; WNND: West Nile Virus neuroinvasive disease; HVLT-R: Hopkins Verbal Learning Test-Revised; BVMT-R: Brief Visuospatial Test-Revised; COWAT: Controlled Oral Word Association Test; TOL: Tower of London; SDMT: Symbol Digits Modalities Test (Oral Version); LNS: Letter-Number-Sequencing subtest; JLO: Judgment of Line Orientation test

^a^Univariate analysis between WNV and WNND groups; Bonferroni-adjusted alpha for statistical significance is 0.0019

Our second objective involved evaluating neuropsychiatric status by duration of illness. Time since infection was grouped into three categories: one month or less (Time 1), six months to two years (Time 2), and two to four years (Time 3). Generally, a greater number of participants demonstrated neuropsychiatric impairment over time, specifically at Time 3 compared to the other time points (Table 3). There were three measures in which the proportion of participants with NP impairment was greater during Time 2 than Time 3; executive functioning (COWAT and Semantic Fluency) and fine motor skills (Grooved—Non-dominant Hand).

**Table 3:** Number of participants with neuropsychiatric impairment by time since infection^a^.

Assessment	Time 1: n = 4	Time 2: n = 28	Time 3: n = 16	Total: n = 49
	n (%)	n (%)	n (%)	n (%)
Verbal Learning and Memory				
HVLT-R Total Recall	2 (50.0)	5 (17.9)	7 (43.8)	15 (30.6)
HVLT-R Delayed Recall	2 (50.0)	3 (10.7)	8 (50.0)	13 (27.1)
HVLT-R Delayed Recognition	2 (50.0)	4 (14.3)	4 (25.0)	10 (20.8)
Visual Learning and Memory				
BVMT-R Total Recall	1 (25.0)	2 (7.1)	2 (12.5)	5 (10.2)
BVMT-R Delayed Recall	0 (0)	2 (7.1)	3 (18.8)	5 (10.2)
BVMT-R Delayed Recognition	1 (25.0)	5 (17.9)	3 (18.8)	9 (18.4)
Executive Functioning				
COWAT	3 (75.0)	10 (35.7)	2 (12.5)	15 (30.6)
Semantic Fluency	0 (0)	5 (17.9)	2 (12.5)	7 (14.3)
TOL—Rule Violation	0 (0)	1 (3.6)	1 (6.3)	2 (5.0)
TOL—Time violation	1 (25.0)	5 (17.9)	5 (31.3)	12 (25.0)
TOL—Execution time	0 (0)	3 (10.7)	2 (12.5)	5 (10.2)
TOL—Problem solving time	0 (0)	5 (17.9)	4 (25.0)	9 (18.4)
Information Processing Speed				
SDMT	0 (0)	2 (7.1)	3 (18.8)	5 (10.2)
Attention				
Stroop Interference	0 (0)	2 (7.1)	2 (12.5)	4 (8.2)
LNS	0 (0)	0 (0)	1 (6.3)	1 (2.0)
Digit Span	0 (0)	0 (0)	0 (0)	0 (0)
Visual-spatial Ability				
JLO	0 (0)	0 (0)	2 (12.5)	2 (4.1)
Fine Motor Skills				
Grooved—Dominant Hand	2 (50.0)	8 (28.6)	7 (43.8)	17 (34.7)
Grooved—Non-dominant Hand	1 (25.0)	9 (32.1)	5 (31.3)	15 (30.6)

WNV: West Nile virus; WNND: Neuroinvasive West Nile virus; HVLT-R: Hopkins Verbal Learning Test-Revised; BVMT-R: Brief Visuospatial Test-Revised; COWAT: Controlled Oral Word Association Test; TOL: Tower of London; SDMT: Symbol Digits Modalities Test (Oral Version); LNS: Letter-Number-Sequencing subtest; JLO: Judgment of Line Orientation test.

^*a*^Time since infection: 1 month or less (Time 1), 6 months to 2 years (Time 2), and 2 to 4 years (Time 3)

## Discussion

### Summary of findings

The current study provides a comprehensive description of neuropsychiatric manifestations of WNV infection that include clinical, laboratory and neuroimaging data. Despite a favorable prognosis with respect to health related quality of life measures, a substantial number of patients demonstrate impairment on tests of motor functioning, verbal learning and memory, and some measures of executive functioning. Severity of neuropsychological impairment was comparable between groups among all measures, and patterns showed more frequent neuropsychological impairment over time, specifically at two to four years since infection compared to the other time points. Severity of depression, anxiety, stress, a fatigue appeared to be within normal ranges and was also comparable between groups.

Our data provide objective evidence of cognitive difficulties among a sample of patients, and the findings suggest that in general these deficits can manifest regardless of severity of WNV infection (WNV fever or WNND). Also, for some individuals these cognitive problems seem to persist for an extended period of time. These findings indicate that the patterns of NP manifestation are unclear, and appear to be systemic rather than a result of neuroinvasive forms of WNV infection specifically.

To date, there has been only one published study that has used standardized, objective neuropsychological tests to assess cognitive performance in patients with WNV infection [19]. Carson and colleagues (2006) conducted a cross-sectional study in North Dakota, USA using a battery of standardized neuropsychological tests that assessed attention, executive functioning, language, learning and memory, motor functioning, verbal intellectual skills, and visual spatial functioning. Depending on the test, assessments of executive function revealed mild to moderate impairments in 7% to 36% of all participants, which included 15 patients with neuroinvasive disease and 34 with non-neuroinvasive disease. Tests of motor function revealed that 34 (69%) had abnormalities in motor speed and in 21 (43%) these deficits were severe. Moderate impairment was also observed among verbal intelligence skills as well as learning and memory [19]. Although this report provides important preliminary data, it lacks serial observations over time.

Our findings are however in agreement with those of this study, with the exception of verbal intelligence, where a greater proportion of participants in the hospitalized group had measurable deficits compared to the non-hospitalized group [19]. We also found no significant differences in motor function between groups, however we observed motor function impairment in 28%-41% of the total sample. Our study is comparable in sample size and environmental setting.

We also found that levels of depression, anxiety, stress, as well as fatigue, were within normal limits, despite earlier studies reporting contradictory findings. This may be attributed to the difference in measurement scales (i.e. DASS vs. CES-D), or to classifications of severity (mild/moderate/severe vs. normal/abnormal) [20].

Taken together, these findings provide evidence for similar mechanisms of NP impairment, despite severity of infection. Contrary to the inherent hypothesis that WNND would result in more severe NP deficits, we observed comparable levels of impairment across all cognitive functioning measures in the entire sample. This is suggestive of a systemic pattern of infection, whereby damage to neurons in the frontal lobe regions that are responsible for cognitive functioning is thought to be caused by the viral infection itself spreading to the brain [73, 74], but perhaps also a result of increased levels of inflammatory markers, leukocytes, and microglia caused by an immune response to the infection [75, 76]. Systemic inflammation has been shown to induce cognitive and behavioral changes and accelerate disease progression in neurodegenerative disorders [77], which may explain how this occurs as a result of WNV infection regardless of neuroinvasive status.

Given that assessment of neuropsychological function among patients with WNND is rare, our study addresses an important area of research. Currently available studies on WNV include few general questions about memory, if at all, with a primary focus of identifying medical, functional, and neurological outcomes rather than neuropsychological status. We also compared different classifications of WNV, which is not commonly done in such studies, and performed serial observations to evaluate disease progression and impairment across time, which is also not extensively reported in the literature.

However, our study was limited by the potential for selection bias, whereby patients with a more severe form of the illness may have been more likely to participate. We have overcome this limitation by enrolling participants using provincial laboratory data (where all testing for WNV is conducted), as well as assessing whether there are any systematic differences between enrolled and non-enrolled patients. There may also be a potential for misclassification of participants based on the definitions set forth by Sejvar et al., as not every participant had an LP measurement available.

### Implications

There are few data describing the long-term neuropsychological outcome of patients with severe WNV infection. We anticipate that data from this study will inform patients and their healthcare providers about the expected course of recovery, and will help prepare patients and caregivers to seek suitable supports beyond the initial recovery period. Additionally, the study data will alert healthcare providers to the need to plan for and implement appropriate interventions (e.g., cognitive rehabilitation) early in the course of recovery so as to minimize the negative impact of persisting cognitive difficulties on daily functioning. It is anticipated that data from this study will be an important component of the knowledge of WNV and flaviviruses in general, and will highlight the important contributions of Canadian research in the field.

We sought to describe the course of neuropsychological functioning following WNV infection in order to establish whether cognitive problems progress or remit over time using a longitudinal study and objective assessment. Studies in this field should continue to explore the neuropsychological effects of WNV, but also seek to understand the mechanisms of the disease and the ambiguous patterns of infection and clinical manifestation. Larger studies may aid in identifying risk factors for adverse outcomes among this population.

## Supporting Information

S1 File This is Primary Data file for this study. (XLSX)
